# Gender, diversity, and the responsible assessment of researchers

**DOI:** 10.1371/journal.pbio.3001036

**Published:** 2021-04-27

**Authors:** Krishma Labib, Natalie Evans

**Affiliations:** Amsterdam University Medical Centers, Vrije Universiteit Amsterdam, Department of Law, Ethics and Humanities, Amsterdam Public Health Institute, Amsterdam, the Netherlands

## Abstract

In response to the Hong Kong Principles for assessing researchers, this Formal Comment argues that it is time to take gender and diversity considerations seriously in the pursuit of fostering research integrity; this requires acknowledging and reshaping the influence of research assessment criteria on researcher representation.

The Hong Kong Principles (HKP) for assessing researchers [1], a product of the 2019 World Conference on Research Integrity, were published in *PLOS Biology* this past July. The principles concern research institutions’ assessment of researchers according to responsible research criteria. The HKP value issues ranging from complete reporting and open science to a diversity of other essential research tasks (e.g., peer reviewing).

We applaud this initiative and believe it is an important step forward because it directly addresses a root cause of many issues that erode research integrity: the unfair reward structures and perverse incentives that researchers encounter [2]. Reforming research assessment practice to reward responsible research, rather than privileging publication volume, is crucial for incentivizing research integrity.

We were surprised that HKP explicitly refrain from considering gender and other issues related to diversity and inclusiveness in researcher assessment. They rather state that, “[t]hese themes require an assessment of a group of researchers (e.g., research institution) when making decisions about funding allocations or human resources policies. Furthermore, these issues concern the social justice and societal relevance of research rather than research integrity.” (p. 9) [1]. We disagree on a number of counts.

First, we challenge the assertion that gender and diversity issues concern social justice and societal relevance of research rather than research integrity. Such a strong distinction between societal relevance and research integrity is difficult to justify; although the field of research integrity was traditionally narrowly defined as pertaining to misconduct issues, it is increasingly acknowledged as addressing general issues of research quality, relevance, and reliability [3]. Furthermore, diversity in research teams is not only important for issues related to social justice and societal relevance, but also crucial for maintaining scientific objectivity and trust in science [4]. Researchers’ backgrounds influence the way that research is funded, conducted, and applied; to prevent science from becoming biased toward certain assumptions and avoid gaps in knowledge, diverse research teams are needed [4]. A lack of diversity in the research community can be detrimental because, through shutting out important perspectives from the research process, it can create undesirable scientific and social effects. For instance, current health research methods commonly entail gender bias, possibly due to the underrepresentation of women in leading research and publishing positions, which not only distorts the public health knowledge base but can also lead to health disparities [4]. Similarly, a lack of early attention and research on the differential impact of the Coronavirus Disease 2019 (COVID-19) on people of different ethnic groups has posed a challenge in curbing mortality and poor health outcomes among Black, Asian, and other ethnic minority groups in several countries such as the United Kingdom and the United States of America [5]. When the research knowledge base is biased in terms of gender or other types of diversity, as is the case with these examples, the trustworthiness of the research itself and its benefit for society are undermined, as it becomes questionable whether the research has employed the right questions and methods to elicit relevant findings for society. Therefore, inclusion of diverse perspectives should not just focus on improving participation of patients and other citizens in research—good practices highlighted in the HKP’s article—but also by improving representation in research teams themselves.

Second, in our view, current researcher assessment practices are funding allocation schemes or human resource policies of research institutions, which affect individual researchers and systematically disadvantage entire groups of researchers, including women and those from a minority background [6]. For instance, the focus on number of publications in researcher assessment disadvantages researchers (mostly female) who need to temporarily take leave to have children [7]. To improve representation in relation to gender and diversity within research teams and departments, it is essential to pay attention to their influence beyond individual assessment performance. The HKP article [1] describes how recognizing other tasks, such as peer review and mentoring, leads to an increase in the number of women promoted (p. 8). Other research suggests that using altmetrics to assess research impact might help narrow the gap between men and women [8]. Hence, the individual assessment of researchers is intimately related to group performance. It is disappointing that the HKP fail to recognize this or to call for attention to the impact of their recommended assessment criteria on diversity issues.

Our plea to the research integrity community is to take gender and diversity considerations seriously, especially in the pursuit of fostering research integrity. This means researcher assessment approaches which acknowledge that systemic disadvantages can be introduced or exacerbated with individual assessment criteria and which contribute toward improving representation within research teams and across seniority levels.
